# Unraveling the Novel Protective Effect of Patchouli Alcohol Against *Helicobacter pylori*-Induced Gastritis: Insights Into the Molecular Mechanism *in vitro* and *in vivo*

**DOI:** 10.3389/fphar.2018.01347

**Published:** 2018-11-22

**Authors:** Da-wei Lian, Yi-fei Xu, Wen-kang Ren, Li-jun Fu, Fang-jun Chen, Li-yao Tang, Hui-ling Zhuang, Hong-ying Cao, Ping Huang

**Affiliations:** ^1^School of Pharmaceutical Sciences, Guangzhou University of Chinese Medicine, Guangzhou, China; ^2^Dongguan & Guangzhou University of Chinese Medicine Cooperative Academy of Mathematical Engineering for Chinese Medicine, Guangzhou University of Chinese Medicine, Guangzhou, China

**Keywords:** patchouli alcohol, *Helicobacter pylori*, gastritis, oxidative injury, inflammasome, gastric epithelial cell

## Abstract

Patchouli alcohol (PA), a natural tricyclic sesquiterpene extracted from *Pogostemon cablin* (Blanco) Benth. (Labiatae), has been found to exhibit anti-*Helicobacter pylori* and anti-inflammatory properties. In this study, we investigated the protective effect of PA against *H. pylori-*induced gastritis *in vitro* and *in vivo*, and determined the underlying mechanism. In the *in vivo* experiment, a C57BL/6 mouse model of gastritis was established using *H. pylori* SS1, and treatments with standard triple therapy or 5, 10, and 20 mg/kg PA were performed for 2 weeks. Results indicated that PA effectively attenuated oxidative stress by decreasing contents of intracellular reactive oxygen species (ROS) and malonyldialdehyde (MDA), and increasing levels of non-protein sulfhydryl (NP-SH), catalase and glutathione (GSH)/glutathione disulphide (GSSG). Additionally, treatment with PA significantly attenuated the secretions of interleukin 1 beta (IL-1β), keratinocyte chemoattractant and interleukin 6 (IL-6). PA (20 mg/kg) significantly protected the gastric mucosa from *H. pylori*-induced damage. In the *in vitro* experiment, GES-1 cells were cocultured with *H. pylori* NCTC11637 at MOI = 100:1 and treated with different doses of PA (5, 10, and 20 μg/ml). Results indicated that PA not only significantly increased the cell viability and decreased cellular lactate dehydrogenase (LDH) leakage, but also markedly elevated the mitochondrial membrane potential and remarkably attenuated GES-1 cellular apoptosis, thereby protecting gastric epithelial cells against injuries caused by *H. pylori*. PA also inhibited the secretions of pro-inflammatory factors, such as monocyte chemotactic protein 1 (MCP-1), tumor necrosis factor-α (TNF-α) and IL-6. Furthermore, after PA treatment, the combination of NACHT, LRR, and PYD domains-containing protein 3 (NLRP3) and cysteine-aspartic proteases 1 (CASPASE-1), the expression levels of NLRP3 inflammasome-related proteins, such as thioredoxin-interacting protein (TXNIP), pro-CASPASE-1, cle-CASPASE-1, and NLRP3 and genes (*NLRP3* and *CASPASE1*) were significantly decreased as compared to the model group. In conclusion, treatment with PA for 2 weeks exhibited highly efficient protective effect against *H. pylori*-induced gastritis and related damages. The underlying mechanism might involve antioxidant activity, inhibition of pro-inflammatory factor and regulation of NLRP3 inflammasome function. PA exerted anti-*H. pylori* and anti-gastritis effects and thus had the potential to be a promising candidate for treatment of *H. pylori*-related diseases.

## Introduction

*Helicobacter pylori* is one of the most popular pathogens because it infects approximately half of the world population (Burucoa and Axon, 2017). Since this bacterial species was recognized in 1984 by Marshall and Warren, *H. pylori* infection and resulting gastritis have become the focus of research (Marshall and Warren, 1984). In 1994, *H. pylori* was categorized by the International Agency of Research on Cancer as type I carcinogen; a number of studies reported that *H. pylori* causes gastric ulcer, gastritis and gastric adenocarcinoma (Kuipers, 1997). Successful eradication of *H. pylori* would effectively relief gastric symptoms and reduce the rate of ulcer recurrence and gastric carcinoma (Wang et al., 2014). Triple therapy (two antibiotics combined with a proton pump inhibitor) is commonly used to clear gastric *H. pylori* infection; however, the efficiency of this treatment had greatly reduced because of the development of bacterial resistance (Connor et al., 2017).

*Helicobacter pylori* could colonize the human gastric epithelium, inducing the release of pro-inflammatory factors and the associated oxidative damage. Pro-inflammatory factors, such as IL-8, trigger and activate neutrophils, which are the major contributor to the production of ROS (Naito and Yoshikawa, 2002). Excessive ROS could lead to the activation of NACHT, LRR and PYD domains-containing protein 3 (NLRP3) inflammasome, which belongs to the nucleotide-binding oligomerization domain family; NLRP3 induces the cleavage of pro-IL-1β, which is then transformed into activated interleukin 1 beta (IL-1β) (Kim et al., 2013; Semper et al., 2014). A previous work reported the strong correlation between overproduction of IL-1β and development of gastric tumor (Huang et al., 2013). Therefore, prevention of ROS generation and IL-1β activation is beneficial to treatment of *H. pylori*-related gastritis and tumors.

*Pogostemon cablin* (Blanco) Benth. (Labiatae) has been used in Asian countries for hundreds of years to clinically treat gastro-intestinal diseases, such as dyspepsia, gastritis as well as ulcer, and patchouli alcohol was one of its major components. Our previous research indicated that PA exerted specific anti-*H. pylori* effect and a wide range of anti-oxidant activities (Zheng et al., 2014; Xu et al., 2017). Scholars have also reported the anti-inflammatory effects of PA against xylene-induced oedema in mouse ear, carrageenan-induced oedema in rat paw and *H. pylori* urease-induced cell cytotoxicity (Li et al., 2011; Xie et al., 2016). In the present study, we designed experiments to investigate the effects of PA on *H. pylori*-induced gastritis and oxidative stress injury in mice. We also determined the potential mechanism by altering NLRP3 activity and using gastric epithelial cells (GES-1) and an *H. pylori* co-culture model.

## Materials and Methods

### Chemicals and Reagents

Columbia agar base and brain heart infusion (BHI) were obtained from OXOID (United States). Sheep blood was acquired from Pingrui Biotechnology (China). Poloxamer 407 was purchased from BASF Chemical Ltd. (Germany). Vancomycin HCl, trimethoprim, cefsulodin sodium, amphotericin, metronidazole (MET) and clarithromycin (CLR) were supplied by Toku-E (Japan). TRIzol Reagent was provided by Life Technologies (United States). FastQuant RT Kit (with gDNAse) and Super Real Pre Mix (SYBR Green) were obtained from Qiagen (Germany). Lactate dehydrogenase (LDH), catalase (CAT), non-protein sulfhydryl (NP-SH), glutathione (GSH)/glutathione disulfide (GSSG), myeloperoxidase (MPO), superoxide dismutase (SOD), and malonyldialdehyde (MDA) kits were acquired from Jiancheng Company (China). CBA kits for interleukin 6 (IL-6), IL-1β and keratinocyte chemoattractant (KC, IL-8) were purchased from BD (United States). IL-6, IL-1β, monocyte chemotactic protein 1 (MCP-1) and tumor necrosis factor-α (TNF-α) ELISA kits and cell apoptosis and cell cycle kits were supplied by 4A Biotech (China). Rhodamine 123 and DCFH-DA were provided by Beyotime^®^ Biotechnology (China). All other reagents used were of analytical grade.

### Drug Preparation

**FIGURE 1:**
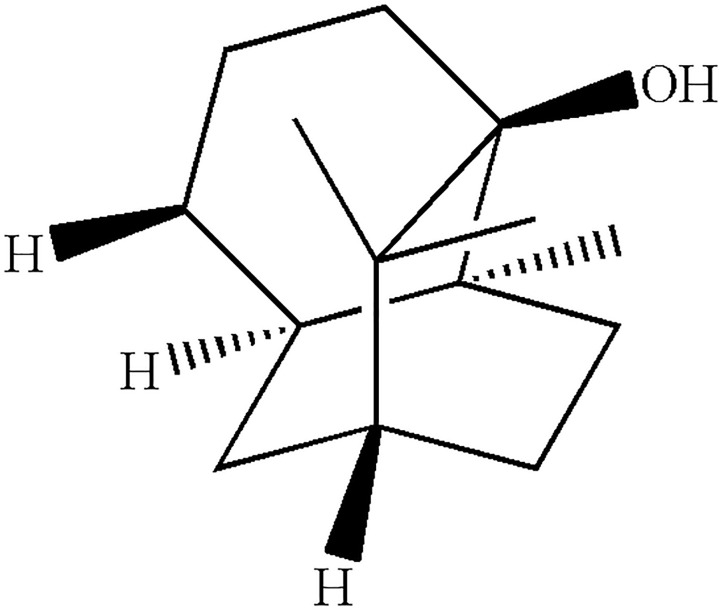
Chemical structure of PA.

Patchouli alcohol (purity > 99%, and the plant material was identified by Prof. Wei Li, Guangzhou University of Chinese Medicine, voucher 13-05-13) was prepared based on our previous publication and further confirmed by melting point, IR, ^1^H and ^13^C NMR and MS analyses (Figure 1) (Qing et al., 2014). In the *in vitro* study, DMSO was used to dissolve PA and served as control (DMSO < 0.5% in all experiments). In the *in vivo* study, poloxamer 407 was used to prepare PA solid dispersion as previously described (Liao et al., 2015). Briefly, PA and poloxamers 407 were mixed at the ratio of 1:5, and melted at 70°C. Then, the mixture was solidified rapidly by dropping into 10°C. After 30 min, diluted water was added to adjust to the final concentration. The doses of PA in this *in vitro* (5, 10, and 20 μg/ml) and *in vivo* (5, 10, and 20 mg/kg) study were adopted for its anti-*H. pylori* (25–75 μg/ml) and anti-ulcer (10, 20, and 40 mg/kg) effects as shown in our previous publication (Zheng et al., 2014; Xu et al., 2017). Besides, the LD_50_ of PA by oral administration in mice was 4.693 g/kg, which was much higher than the effective doses (He et al., 2011).

### *H. pylori* Strains and Growth Condition

NCTC11637 was obtained from the American Type Culture Collection. Sydney Strain-1 (SS1) was kindly provided by Prof. Richard Ferrero of Monash University, Australia. *H. pylori* was stored in BHI supplemented with 30% glycerol at −80°C. *H. pylori* were then cultured in Columbia agar with 7% sheep blood (or BHI with 10% FBS) in a tri-gas incubator (Nuaire Nu-5831E, United States) at 37°C under 10% CO_2_, 5% O_2_ and 85% N_2_ for 48–72 h. The culture was added with Dent’s medium (10 mg/l vancomycin, 5 mg/l trimethoprim, 5 mg/l cefclidine, and 5 mg/l amphotericin B) to avoid contamination.

### Animals

Five- to six-week-old male C57BL/6 mice were purchased from Charles River (Beijing, China). Only male mice were included in the experiment because of their higher successful modeling rate than female mice. The mice were reared under controlled conditions (22 ± 2°C and relative humidity of 50 ± 5%) under a 12 h light/dark cycle and given free access to food and water. The mice were fed with standard lab mice diet (Growing Feed After Irradiation) purchased from Guangdong Medical laboratory Animal Centre. This study was carried out in accordance with the recommendations of NIH Guide for the Care and Use of Laboratory Animals, and the protocols were approved by the Animal Experimental Ethics Committee of Guangzhou University of Chinese Medicine (NO. S2016082, Guangzhou, China).

### Establishment and Treatment of *H. pylori*-Related Gastritis Model

A mouse model of *H. pylori* infection was established using the method reported in our published work (Xu et al., 2017). *H. pylori* SS1 strain was passaged twice in Columbia agar, inoculated in BHI (10% FBS) at McFarland 0.1 and shaken at 120 rpm for 16–20 h. Logarithmic-phase bacteria (1.5 × 10^7^ CFU/ml, 0.25 ml) were orally gavaged into the mice by using a 24-gauge needle three times at 2-day interval over a period of 5-days. The mice were fasted for 12 h before gavage and resumed diet after 2 h. Salt (2%) was added to the drinking water to increase bacterial virulence. After 1 week, PCR and rapid urease test were performed to examine *H. pylori* infection. The remaining mice were further fed for 10 weeks and randomly divided into the following groups: control (sterile BHI + poloxamer 407), model (*H. pylori* + poloxamer 407), triple therapy (*H. pylori* + omeprazole 400 μmol/kg/day, MET 14.2 mg/kg/day and CLR 7.15 mg/kg/day) and PA (5, 10, and 20 mg/kg) groups. After 2 weeks of oral administration, the mice were anesthetised. Whole blood was collected from the orbit and centrifuged to obtain serum. The mice were then sacrificed. The pylorus and antrum of the stomach were isolated and divided into two parts. One part was fixed in 4% paraformaldehyde and stained with hematoxylin-eosin (HE) and boracic acid methylene blue (BAMB). The other part was homogenized in Tris buffer (20 mM, pH 7.5) on ice and then was centrifuged at 12,000 *g* at 4°C for 10 min. The supernatants were used for measurement of oxidative indices, such as CAT, GSH, MPO, NP-SH, SOD and MDA. The concentration of protein in the supernatants was measured by the Bradford method. Besides, the pro-inflammatory factors (IL-6, KC, and IL-1β) in serum were detected by flow cytometry using a CBA kit. The experiment procedures were conducted according to the manufacturer’s instructions.

### Cell Culture and Treatment

GES-1 cells were kindly provided by the First Affiliated Hospital of Guangzhou University of Chinese Medicine. The cells were cultured in high-glucose DMEM medium supplemented with 10% FBS at 37°C in a 5% CO_2_ incubator. The cells were passaged when they reached 80% confluency. The cells were then divided into five groups: control (DMSO), model (DMSO + *H. pylori*) and PA 5, 10 and 20 (5, 10, and 20 μg/ml PA + *H. pylori*) groups. In the PA groups, GES-1 cells were cocultured with *H. pylori* at MOI = 100 in a cell incubator and treated with different concentrations of PA for 24 h.

### Evaluation of GES-1 Cell Viability After Incubation With *H. pylori*

The cell viability of GES-1 cell exposed to *H. pylori* and PA was evaluated by determining the percentage of LDH leakage and trypan blue assay. The supernatant and remaining GES-1 cells were collected after incubation with *H. pylori* at MOI = 100 with or without PA treatment (5, 10, and 20 μg/ml) for 24 h. DMSO served as control. LDH was detected according to the instruction manuals. Cell viability was assessed by counting trypan blue-negative cells.

### GES-1 Cell Mitochondrial Membrane Potential (MMP) and ROS Generation Assay

GES-1 cells were treated as described above, washed three times with PBS and incubated with 1 μM rhodamine 123 for 20 min. The cells were washed again with ice-cold PBS three times and divided into two parts. Fluorescence intensity was measured on one part through fluorescence microscopy, and the results were expressed as fluorescence intensity per cell. The other part was digested using 0.25% trypsin, washed three times with ice-cold DMEM solution to remove the remaining fluorescence dye and subjected to flow cytometry for MMP analysis at excitation and emission wavelengths of 507 and 529 nm, respectively. ROS generation assay was performed using the modified MMP detection method. DCFH-DA (1:1000), instead of rhodamine 123, was used for staining to measure intracellular ROS. The excitation and emission wavelengths used in flow cytometry were adjusted to 488 and 525 nm, respectively.

### GES-1 Cell Apoptosis and Pro-inflammatory Factor Production Assay

GES-1 cells were treated as described above and centrifuged. The supernatant was removed and used for ELISA assay to investigate the generation of IL-6, IL-1β, MCP-1, and TNF-α with or without PA treatment. ELISA assay was performed according to the manufacturer’s instructions of the kits. Adherent cells were washed with PBS, digested using 0.25% trypsin (without EDTA) and resuspended in 500 μl of PBS containing 5 × 10^5^ cells. The cells were then added with 5 μl of annexin V-FITC, incubated for 20 min and gently mixed with 5 μl of PI. The apoptosis of GES-1 cells was immediately analyzed through flow cytometry.

### RNA Isolation and Real-Time Quantitative PCR

Total RNA was isolated from the cells by using Trizol reagent, and the concentrations of the extracted RNA were measured spectrophotometrically at 260 nm. RNA quality was assessed based on the absorbance ratio at 260 and 280 nm. A260/A280 values ranging from 1.9 to 2.1 were considered acceptable. The total RNA was reverse transcribed into cDNA by using a PrimeScript RT reagent kit with gDNA eraser. Real-time PCR analysis was performed using SYBR Green according to the manufacturer’s instructions. The reactions were run at 50°C for 2 min and 95°C for 10 min, followed by 45 cycles at 95°C for 15 s and 60°C for 1 min on the Applied Biosystems Step-One Fast Real-Time PCR system (Table 1). The results were quantified using 2^−ΔΔCT^ method.

**Table 1:** Primer sequences.

Gene	Forward primer (5′-3′)	Reverse primer (5′-3′)
*NLRP3*	GGAGAGACCTTTATGA GAAAGCAA	GCTGTCTTCCTG GCATATCACA
*CASPASE1*	GGAAACAAAAG TCGGCAGAG	ACGCTGTACCC CAGATTTTG
*ASC*	CAGCCAAGCCAGGCC TGCACTTTAT	TTGCTTGGG TTGGTGGGCTCG
*GADPH*	CCACATCGCT CAGACACCAT	GGCAACAATATCC ACTTTACCAGAGT

### Western Blot Analysis

Total protein was extracted using RIPA buffer and centrifuged at 10,000 *g* and 4°C for 15 min. The supernatant was collected and subjected to BCA Protein Assay kit to measure protein concentration. Cell homogenates were denatured with reducing Laemmli SDS sample buffer and boiled in a metal bath for 5 min at 95°C. Equal amounts of protein samples were separated by 12% SDS–PAGE and transferred onto PVDF membrane. The membrane was incubated in primary antibodies at 4°C overnight and treated with anti-rabbit IgG (1:2000) or anti-mouse IgG (1:2000) for 2 h at room temperature. The primary antibodies were anti- thioredoxin-interacting protein (TXNIP, 1:1000, Cell Signal Technology), anti-NLRP3 (1:1000, Cell Signal Technology), anti-CASPASE-1 (8:5000, Santa Cruz) and anti-ASC (8:5000, Santa Cruz). Anti-β-ACTIN (1:1000, BOSTER) was used as internal control. Target bands were detected and analyzed using Image J software (NIH, United States).

### Immunofluorescence Microscopic Analysis

GES-1 cells that adhered to the round glass coverslips were fixed in 4% buffered paraformaldehyde and permeabilised with 0.1% Triton X-100. The cells were then incubated using goat anti-NLRP3 (1:200, Abcam), mouse anti-CASPASE-1 (1:200, Santa Cruz) and rabbit anti-ASC (1:100, Santa Cruz). After incubation with primary antibodies, the samples were washed and labeled with corresponding Alexa Fluor-488- and Alexa Fluor-555-conjugated secondary antibodies. The cells were visualized under a Zeiss LSM800 microscope. Colocalization was analyzed by Image Pro Plus software. Colocalization coefficient was represented by Pearson’s correlation coefficient as described.

### Statistical Analysis

Results were expressed as mean ± SEM and analyzed using SPSS 21.0. LSD test or Dunnett’s test was used for multiple groups based on homogeneity of variance. Mann–Whitney test was adopted for enumerated data. *P <* 0.05 indicated statistical significance.

## Results

### Effect of PA on *H. pylori-*Related Gastritis

**FIGURE 2:**
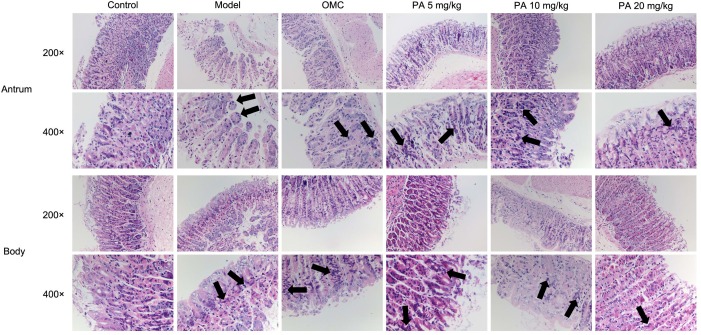
Histological evaluation on the effect of PA on the gastric antrum and body in *Helicobacter pylori*-infected gastritis (hematoxylin-eosin staining; magnification: 200× or 400×) and back arrows points to inflammatory cell infiltration. BL/6 mice were infected with *H. pylori* SS1 strain for 10 weeks and treated with vehicle (model group), triple therapy (omeprazole + MET +CLR, positive group), and different doses of PA (5, 10, and 20 mg/kg groups) for 2 weeks. The control group received 2 weeks of vehicle treatment without *H. pylori* challenge. (*n* = 8).

**FIGURE 3:**
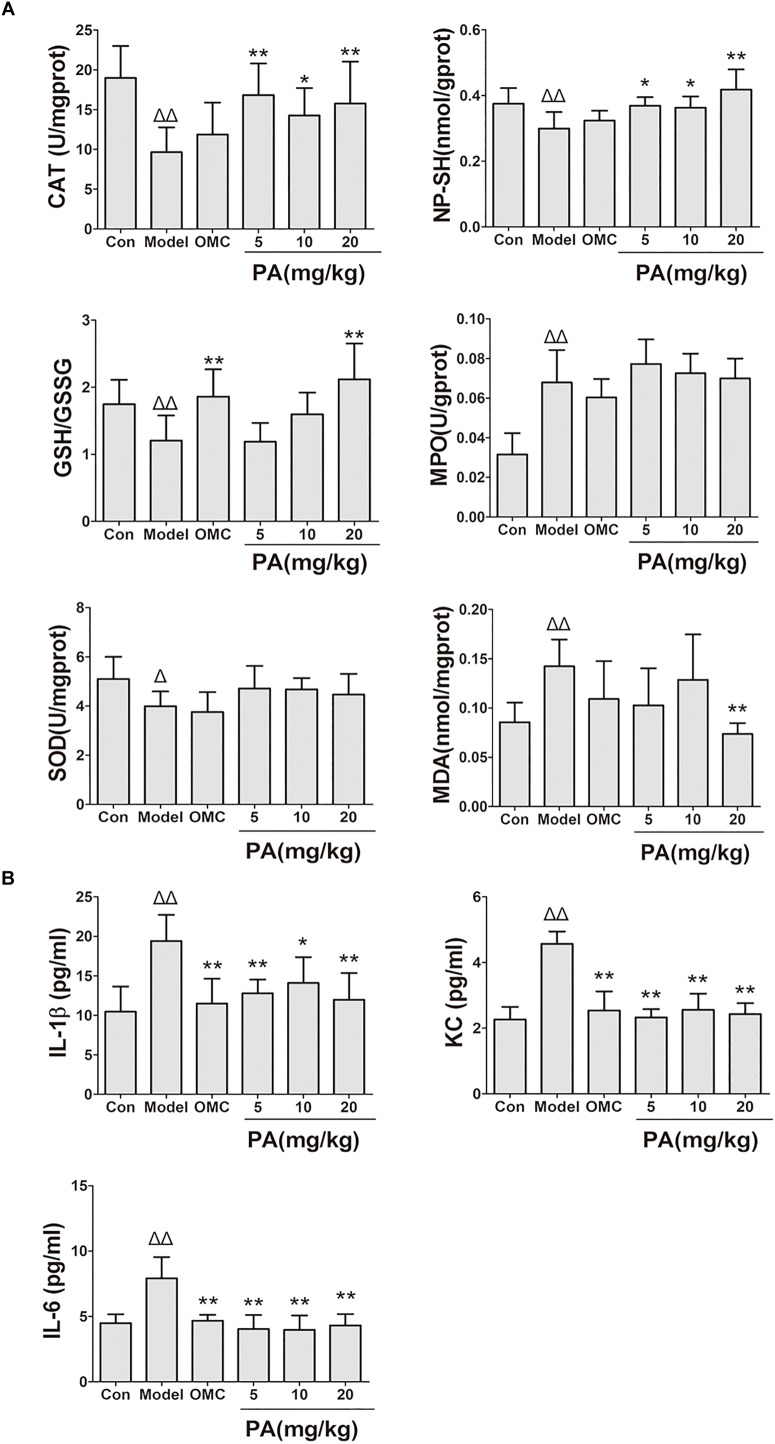
Effects of PA on gastric oxidative factor levels and pro-inflammatory cytokine expression in *Helicobacter pylori*-infected mice. C57BL/6 mice were infected with *H. pylori* SS1 strain for 10 weeks and treated with vehicle (model group), triple therapy (omeprazole + MET +CLR, positive group), and different doses of PA (PA 5, 10, and 20 mg/kg groups) for 2 weeks. The control group received vehicle treatment for 2 weeks without *H. pylori* challenge. After the treatment, the gastric antrum and pylorus were removed, homogenized and centrifuged to obtain the supernatant. The supernatant was quantified by BCA assay and stored at −80°C for further detection. **(A)** Oxidant factor levels (CAT, MPO, and SOD activities; NP-SH and MDA contents; GSH/GSSG). **(B)** Pro-inflammatory cytokine expression levels (IL-1β, KC, and IL-6). Δ*P <* 0.05, ΔΔ*P <* 0.01 compared with the control group. ^∗^*P <* 0.05, ^∗∗^*P <* 0.01 compared with the model group (*n* = 8).

*Helicobacter pylori* infection was confirmed by PCR after RUT and BAMB staining (BAMB results were shown in Supplementary Figure S1). The effect of PA on *H. pylori*-related gastritis models was evaluated through HE staining. As shown in Figure 2 and Tables 2, 3, chronic gastritis phenomena, including monocyte aggregation and infiltration and hyperblastosis, were present in the gastric antrum and body of the model groups (Talebkhan et al., 2009). Moreover, the chronic degree of inflammation in the gastric antrum or body significantly decreased by treatment with 20 mg/kg PA (*P <* 0.05, Mann–Whitney test). The assays on oxidative index and pro-inflammatory factors showed significant alterations including increase in MPO, MDA, IL-1β, KC, and IL-6 levels and decrease in SOD, GSH/GSSG, SH-NP, CAT and PGE2 levels in the model mice (Figures 3A,B). The SH-NP content and the CAT activity were enhanced by PA treatment (5, 10, and 20 mg/kg) compared with those in the model group. The MDA content decreased, and the GSH/GSSG level increased in the PA (20 mg/kg) group. The MPO and SOD activities did not significantly change. Triple therapy significantly increased the GSH/GSSG level. Moreover, the levels of IL-1β, KC, and IL-6 decreased with PA treatment (5, 10, and 20 mg/kg; *P <* 0.01 or *P <* 0.05). Triple therapy inhibited the secretion of pro-inflammatory factors (IL-1β, KC, and IL-6).

**Table 2:** Effect of PA on *H. pylori*-induced gastritis in antrum.

	Cases (*n*)	Chronic degree of inflammation in gastric antrum				^a^*P*	^b^*P*
		0	1	2	3		
Control group	8	8	0	0	0		
Model group	8	0	8	0	0	<0.01	
OMC group	8	2	6	0	0		
PA 5 mg/kg	8	0	8	0	0		
PA 10 mg/kg	8	1	7	0	0		
PA 20 mg/kg	8	4	4	0	0		<0.05

*^a^P compared with the control group; ^b^P compared with the model group.*

**Table 3:** Effect of PA on *H. pylori*-induced gastritis in body.

	Cases (*n*)	Chronic degree of inflammation in gastric body xsxs				^a^*P*	^b^*P*
		0	1	2	3		
Control group	8	8	0	0	0		
Model group	8	0	8	0	0	<0.01	
OMC group	8	1	7	0	0		
PA 5 mg/kg	8	1	7	0	0		
PA 10 mg/kg	8	0	8	0	0		
PA 20 mg/kg	8	4	4	0	0		<0.05

*^a^P compared with the control group; ^b^P compared with the model group.*

### Effect of PA on Cell Viability After Incubation With *H. pylori*

**FIGURE 4:**
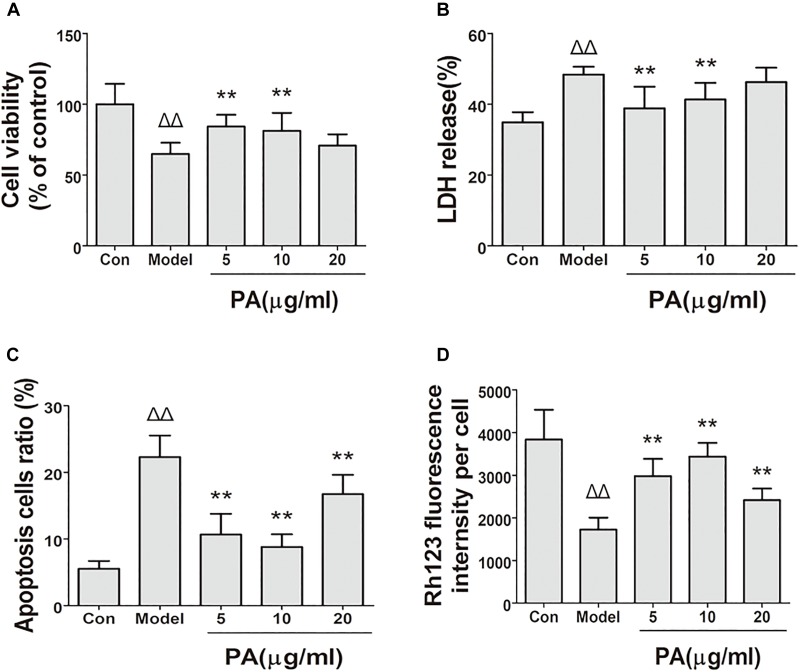
Effects of PA on cell biological activity in *Helicobacter pylori*-infected GES-1 cell. GES-1 cells were infected with *H. pylori* NCTC11637 at MOI = 100:1 for 24 h and treated with DMSO (model group) and 5, 10, and 20 μg/ml PA. Cells in the control group were treated with the same volume of DMSO without *H. pylori* infection. After treatment, the supernatant was obtained for LDH detection, and the rest of the cells were stained with fluorochrome for apoptosis and MMP investigation. **(A)** Cell viability. **(B)** LDH leakage. **(C)** Apoptosis rate in *H. pylori*-infected GES-1 cell treated with PA was assessed by flow cytometry after staining with FITC-conjugated annexin V and propidium iodide. **(D)** Rhodamine 123 was used as a measure of membrane polarization in live cell assays within mitochondria, MMP (stained with rhodamine 123 fluorescence intensity) of *H. pylori*-infected GES-1 cell. Δ*P <* 0.05, ΔΔ*P <* 0.01 compared with the control group. ^∗^*P <* 0.05, ^∗∗^*P <* 0.01 compared with the model group (*n* = 3).

The protective effect of PA on GES-1 cells infected with *H. pylori* at MOI = 100 was investigated in terms of the percentage leakage of LDH and trypan blue assay at 24 h (Figures 4A,B). The plasma membrane damage in GES-1 cells increased (48.4%) and the number of viable cells decreased (58.2%) after *H. pylori* incubation compared with those in the control group. Treatment with 10 and 20 μg/ml PA reduced the plasma membrane damage (38.8 and 41.3%, respectively) and cell death (80.2 and 75.2%, respectively; *P <* 0.01 or *P <* 0.05).

### Effect of PA on the Apoptosis of *H. pylori*-Infected Cells

The apoptosis of *H. pylori*-infected GES-1 cell with or without PA was assessed by flow cytometry after staining with FITC-conjugated annexin V and propidium iodide. As depicted in Figure 4C, the cells were divided into two states: viable cells (annexin V−PI−) and apoptotic cells (annexin V+PI±). The number of apoptotic cells increased in the *H. pylori*-infected group (22.3%) compared with that in the control group (5.4%). PA treatment (5, 10, and 20 μg/ml) significantly reduced *H. pylori*-induced cellular apoptosis (10.8, 9.3, and 15.3%, respectively; *P <* 0.01).

### Effect of PA on Cellular MMP in *H. pylori*-Infected Cells

Rhodamine 123 dye could accumulate in mitochondrial membranes in a membrane polarization-dependent manner. GES-1 cells in the control group displayed a bright green fluorescence (Figure 4D). This fluorescence was clearly attenuated when the cells were infected with *H. pylori* for 24 h. By contrast, PA treatment (5, 10, and 20 μg/ml) restored the green fluorescence (*P* < 0.01), suggesting that PA can preserve cellular MMP and protect GES-1 cells from *H. pylori*-induced mitochondrial damage.

### Effect of PA on Cellular Cytokine Production in *H. pylori*-Infected Cells

**FIGURE 5:**
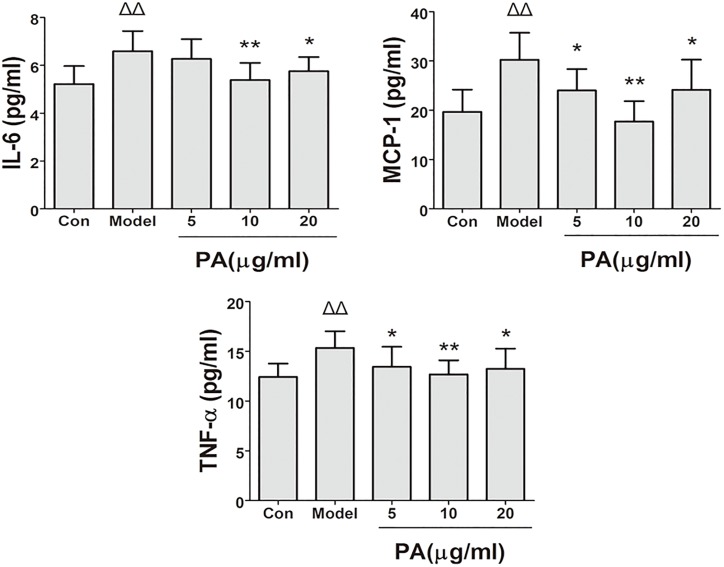
Effects of PA on pro-inflammatory cytokine expression in *Helicobacter pylori*-infected GES-1 cell. GES-1 cells were infected with *H. pylori* NCTC11637 at MOI = 100:1 and treated with DMSO (model group) and 5, 10, and 20 μg/ml PA. Cells in the control group were treated with the same volume of DMSO without *H. pylori* infection. After 24 h of incubation, the supernatant was obtained for detection of pro-inflammatory cytokines (IL-6, TNF-α, and MCP-1). Δ*P <* 0.05, ΔΔ*P <* 0.01 compared with the control group. ^∗^*P <* 0.05, ^∗∗^*P <* 0.01 compared with the model group (*n* = 3).

The effects of PA on the production of cytokines (IL-6, TNF-α, and MCP-1) in *H. pylori*-infected GES-1 cell was shown in Figure 5. Treatment with 5, 10, and 20 μg/ml PA significantly reduced the production of TNF-α and MCP-1. Besides, treatment with 10 and 20 μg/ml PA significantly reduced the production of IL-6 in infected cells (*P <* 0.01 or *P <* 0.05).

### Effect of PA on Cellular ROS Production in *H. pylori*-Infected Cells

**FIGURE 6:**
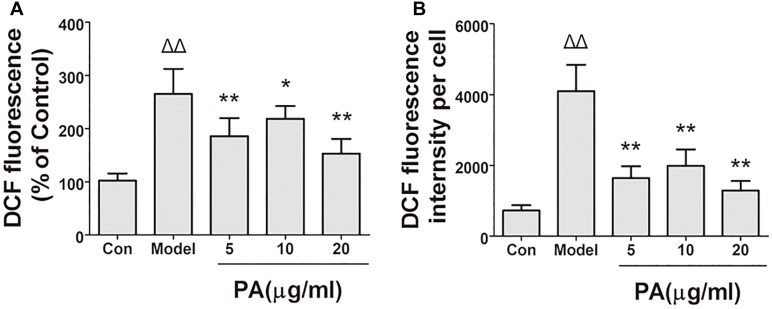
Effects of PA on ROS generation in *Helicobacter pylori*-infected GES-1 cell. GES-1 cells were infected with *H. pylori* NCTC11637 at MOI = 100:1 and treated with DMSO (model group) and 5, 10, and 20 μg/ml PA. Cells in the control group were treated with the same volume of DMSO without *H. pylori* infection. After 24 h of incubation, GES-1 cells were stained with DCFH-DA (1:1000) for 20 min. Fluorescence intensity of the first part of the cells was measured through microscopy. The remaining cell part was digested using 0.25% trypsin and subjected to flow cytometry. **(A)** Quantitative analysis of DCFH-DA accumulation by fluorescence microscope. **(B)** Quantitative analysis of DCFH-DA accumulation by FACS. Δ*P <* 0.05, ΔΔ*P <* 0.01 compared with the control group. ^∗^*P <* 0.05, ^∗∗^*P <* 0.01 compared with the model group (*n* = 3).

The effects of PA on *H. pylori*-induced ROS production are shown in Figure 6. Intracellular ROS was measured using DCFH-DA reagent. The results of the microscopic and flow cytometric analyses illustrated that ROS production was elevated in GES-1 cells infected with *H. pylori* for 24 h. Treatment with PA (5, 10, and 20 μg/ml) significantly attenuated *H. pylori*-induced ROS production (*P* < 0.01). Hence, PA inhibited *H. pylori*-induced oxidative stress in GES-1 cells.

### Effect of PA on *H. pylori*-Induced NLRP3 Inflammasome Formation and Activation

**FIGURE 7:**
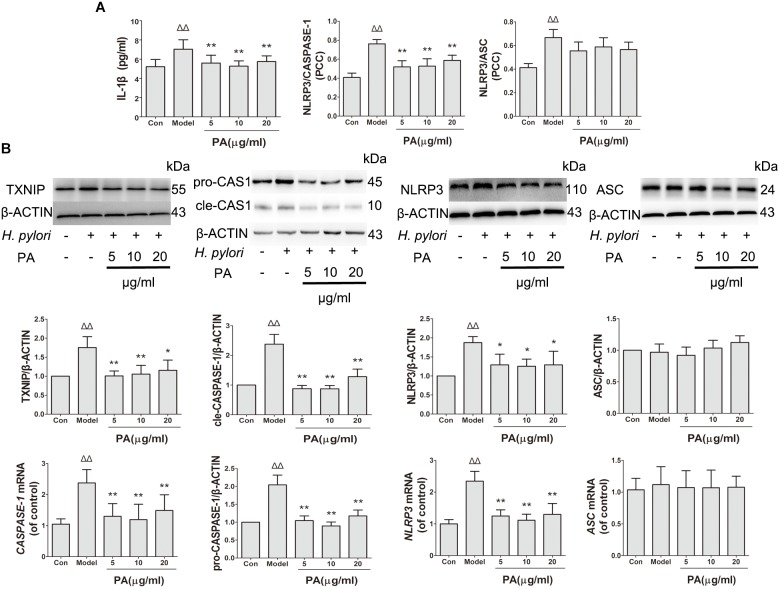
Effects of PA on NLRP3 inflammasome formation and activation in *Helicobacter pylori*-infected GES-1 cell. GES-1 cells were infected with *H. pylori* NCTC11637 at MOI = 100:1 and treated with DMSO (model group) and 5, 10, and 20 μg/ml PA. Cells in the control group were treated with the same volume of DMSO without *H. pylori* infection. After 24 h of incubation, GES-1 cells were fixed in paraformaldehyde for immunofluorescence assay or extracted using RIPA buffer for Western blot assay. **(A)** Quantitative data of the co-localisation efficiency (Pearson’s correlation coefficient) and IL-1β expression level revealed the activated state of inflammasome. **(B)** Western blot analysis showed the effect of PA on the protein expression levels of TXNIP, pro-CASPASE-1 (pro-CAS1)/cleaved CASPASE-1 (cle-CAS1), NLRP3 and ASC. RT-qPCR analysis showed the effect of PA on the gene expression levels of *CASPASE-1*, *NLRP3*, and *ASC*. Quantitative data of Western blot and RT-qPCR analyses are also presented. Δ*P <* 0.05, ΔΔ*P <* 0.01 compared with the control group. ^∗^*P <* 0.05, ^∗∗^*P <* 0.01 compared with the model group (*n* = 3).

This study determined the effects of PA on *H. pylori*-induced NLRP3 inflammasome production and activation in GES-1 cells. Western blot and RT-qPCR analyses were conducted to analyze the inhibitory effect of PA on *H. pylori-*induced expression of inflammasome-related genes (*NLRP3*, *ASC* and *CASPASE-1*) and proteins (NLRP3, ASC, pro-CASPASE-1, cle-CASPASE-1, and TXNIP) (Figure 7B). *H. pylori* stimulation upregulated the gene expression levels of *NLRP3* and *CASPASE-1* and the protein levels of NLRP3, pro-CASPASE-1, cle-CASPASE-1, and TXNIP; moreover, the gene and protein expression levels of *ASC* were comparable after *H. pylori* challenge (*P* > 0.05). After PA treatment, the expression levels of *NLRP3* and *CAPASE-1* and protein levels of NLRP3, pro-CASPASE-1, cle-CASPASE-1, and TXNIP decreased (*P* < 0.05). We then examined the activation of NLRP3 inflammasome by analyzing the co-localisation of NLRP3 inflammasome, CASPASE-1 and ASC (Figure 7A). The colocalization of NLRP3 (red) and CASPASE-1 (green) decreased in the 5, 10, and 20 μg/ml PA treatment groups compared with that in the model group. Similar results were observed in *H. pylori*-induced NLRP3 (red) and ASC (green) over-colocalization, although the effect was not significant. Moreover, PA reduced the release of IL-1β in infected cells, indicating that *H. pylori*-induced NLRP3 inflammasome activation was inhibited by PA treatment.

## Discussion

Novel drugs for treatment of *H. pylori*-related diseases must be developed given that this bacterial species has developed antibiotic resistance. *H. pylori* infection has been speculated to promote gastritis development; the International Agency for Research on Cancer estimated that 6.2% of all cancers are attributed to *H. pylori* (Plummer et al., 2015). The induction of gastric diseases by *H. pylori* has been speculated to begin from non-atrophic gastritis; this condition then develops into atrophic gastritis, intestinal metaplasia, dysplasia and finally to carcinoma and is accompanied with oxidative stress and inflammatory damage to gastric mucosa (Liu et al., 2016). However, successful eradication of bacteria *in vivo* could not prevent the development of several *H. pylori*-related gastric diseases, such as metachronous gastric cancer (Kawanaka et al., 2016). In our previous work, we reported that PA could clear *H. pylori* infection *in vivo* (Xu et al., 2017). The present study demonstrated that PA exhibited protection against *H. pylori-*related gastritis *in vivo* through its antioxidant and anti-inflammatory activities.

*Helicobacter pylori* induces humoral and cellular immune responses, which contribute to bacterial eradication. However, *H. pylori* has developed several mechanisms to avoid being recognized by the immune system of its host (Blaser and Atherton, 2004; Basso et al., 2010; Lina et al., 2014). Various endogenous stress factors, such as ROS or reactive nitrogen species (RNS), are secreted by immune cells, and accumulate in infected tissues (Brandt et al., 2005; Wang et al., 2006; Handa et al., 2010). When *H. pylori* infection continues, the condition of the infected gastric tissue is aggravated, resulting in chronic gastritis. To investigate the effect of PA on *H. pylori*-related chronic gastritis, we first established a mouse model of *H. pylori*-related chronic gastritis by using a previously reported method (Xu et al., 2017). The histopathological alteration suggested that PA could inhibit gastritis and eradicate *H. pylori* in the gastric antrum and body. The anti-inflammatory effect of triple therapy conducted for 2 weeks was weaker than that of PA but still showed high-efficiency *H. pylori* eradication. Treatment with PA also suppressed the gastric antioxidant levels and cytokine expression levels. We prove that PA restored CAT, GSH and NP-SH function, which scavenge superoxide. Interesting, our data show that both CAT and NP-SH function were restored to near normal level at all doses of PA, meanwhile OMC have not effect. Whereas GSH function was only restored at high dose of PA or OMC. There is a close relationship between GSH and *H. pylori* infection (Shirin et al., 2001), so we assume that PA and OMC restore the function of GSH through eradicating *H. pylori.* Furthermore, there is a significant anti-inflammatory effect at all doses of PA and OMC. MDA results from lipid peroxidation of polyunsaturated fatty acids, which can be used to measure the membrane lipid peroxidation. In our research, MDA significantly decreases at high dose of PA but has faint alterations under the treatment of OMC. In summary, we can speculate that the mechanism of PA in treatment of *H. pylori*-related gastritis works in two ways. On the one hand, PA could eliminate *H. pylori* in model mice. On the other hand, the anti-inflammatory effect of PA is possibly associated with reduced secretion of pro-inflammatory cytokines and relief of oxidative stress level.

During infection, *H. pylori* immediately adheres to the gastric epithelial cells and release virulence factors, such as CagA, VacA, and urease (Paniagua et al., 2009; Wang et al., 2009). These factors not only activate the hose immune system but also cause inflammatory reaction or even death of gastric epithelial cells (Calvino-Fernandez et al., 2008; Niwa et al., 2010). In particular, ROS, which is generated from NADPH oxidase in gastric epithelial cells, is speculated to be involved in gastric mucosal chronic infectious diseases by stimulating the release of virulence factors (Nagata et al., 1998; Handa et al., 2007). To investigate the anti-inflammatory mechanism of PA in *H. pylori*-infected diseases, we utilized a coculture model of *H. pylori* and epithelial cells. We first analyzed and compared the function of infected cells during PA intervention. The cell survival rate increased, whereas the apoptosis rate significantly decreased; mitochondrial activity was recovered after PA treatment. Moreover, the survival rate was not elevated during MC treatment (Supplementary Figure S2). The following experiments demonstrated that PA exerts anti-inflammatory effect by inhibiting the production of oxidative stress induced by pro-inflammatory factors and ROS *in vitro*. To further investigate the mechanism of the anti-inflammatory effect of PA, we studied NLRP3 inflammasome.

NLRP3 inflammasome, which belongs to the family of nucleotide-binding and oligomerization domain-like receptors (NLRs), plays a critical role in the pathogenesis of *H. pylori*-related gastritis (Li et al., 2015; Perez-Figueroa et al., 2016). NLRP3 inflammasome assembly and activation involves a two-step mechanism; the primary signal from the activation of toll-like receptors by the lipopolysaccharide of *H. pylori* is responsible for the upregulation of NLRP3 inflammasome-related components, such as NLRP3 and pro-IL-1β, by activating the NF-κB pathway (Bauernfeind et al., 2009). The secondary signal from multiple pathways, including ROS production from *H. pylori*-induced oxidative stress, induces the release of TXNIP from oxidized TRX and the binding of TXNIP to NLRP3, thereby promoting the assembly and oligomerization of inflammasome (Zhou et al., 2010); this phenomenon leads to recruitment of ASC and proteolysis of pro-caspase-1 into cle-CASPASE-1 (cleaved form) to release mature IL-1β (Davis et al., 2011; He et al., 2016). Activation of NLRP3 inflammasome induces inflammation-related cellular dysfunction, pyroptosis or death (Lamkanfi, 2011). Our results suggest that PA reduces NLRP3 and CASPASE-1 protein production. Considering the previous finding that PA inhibits the NF-κB pathway (Jeong et al., 2013), we investigated the gene expression levels of *NLRP3* and *CASPASE-1*. We found that PA significantly reduced the expression levels of these two genes as predicted. Therefore, we speculated that one of the reasons for the inhibition of NLRP3 inflammasome activation by PA is related to blocking the primary signal by inhibiting the formation of inflammasome-related proteins. Furthermore, we found that TXNIP was downregulated and the ROS production decreased; the regional colocalization of NLRP3 and CASPASE-1 was reduced, indicating that the effects of PA might be associated with suppression of the secondary signal by inhibiting NLRP3 inflammasome assembly and constitution. Finally, we confirmed the hypothesis that PA could inhibit NLRP3 inflammasome activation based on the detected levels of activated CASPASE-1 and IL-1β.

We confirmed that PA exerted anti-inflammatory effect on *H. pylori*-infected model *in vitro* and *in vivo*. Combined with our previous finding on PA, the present results show that the anti-inflammatory mechanism of PA acted in two ways; on the one hand, PA eliminated *H. pylori* (Xu et al., 2017) and inhibited bacterial virulence factors expression, such as urease (Yu et al., 2015); on the other hand, it exerted anti-inflammatory activity via the NF-κB pathway (Jeong et al., 2013) and NLRP3 inflammasome activation signaling. Whether these two ways exert mutual influence requires further research. Overall, our findings indicated that PA exhibits immense potential and specific therapeutic value in *H. pylori*-infected gastritis and in the development of innovative antimicrobial drugs.

## Author Contributions

Hy-C, PH, and Yf-X designed the experiments. Dw-L, Wk-R, Lj-F, and Hl-Z performed all the assays. Fj-C and Ly-T analyzed and summarized the data. Yf-X and Dw-L wrote the paper. All the authors gave final approval.

## Conflict of Interest Statement

The authors declare that the research was conducted in the absence of any commercial or financial relationships that could be construed as a potential conflict of interest.
