# Supplementary material for: Unraveling the Novel Protective Effect of Patchouli Alcohol Against Helicobacter pylori-Induced Gastritis: Insights Into the Molecular Mechanism in vitro and in vivo
Source: Front Pharmacol. 2018 Nov 22;9:1347. doi: 10.3389/fphar.2018.01347 (PMC6262355; doi:10.3389/fphar.2018.01347)
Supplement: Supplementary file 1 [file Presentation_1.PDF]

Supplementary Material

Unravelling the novel protective effect of patchouli alcohol against *Helicobacter pylori*-induced gastritis: Insights into the molecular mechanism *in vitro* and *in vivo*

Da-wei Lian, Yi-fei Xu, Wen-kang Ren, Li-jun Fu, Fang-jun Chen, Li-yao Tang, Hong-ying Cao*, Ping Huang *

*** Correspondence:** Hong-ying Cao: 1171629708@qq.com

*** Correspondence:** Ping Huang: hping331@126.com

1 Identification of Helicobacter pylori infection

The gastric antrum and body tissues of *H. pylori*-infected mice were isolated and stained with boracic acid methylene blue. Fig. S1 showed the results for the control and *H. pylori*-infected groups. *H. pylori* colonised the surface of gastric epithelial cells (brown arrow).

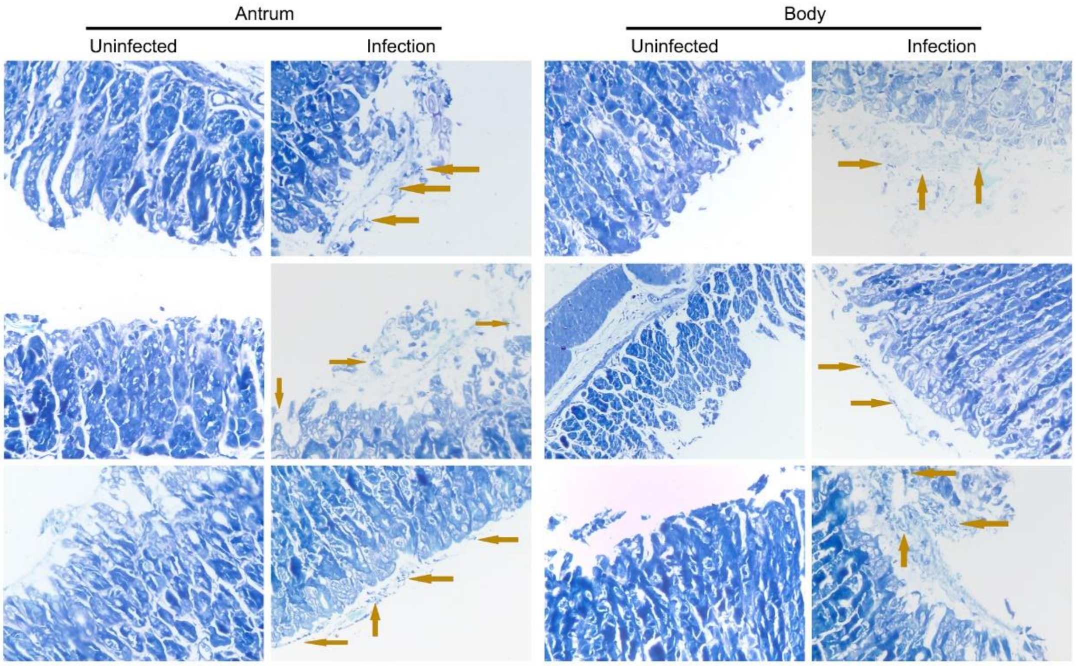

Supplementary Figure 1. Boracic acid methylene blue (BAMB) staining for the detection of *Helicobacter pylori* in the gastric antrum and body tissues. (BAMB, 400×, n=8)

2 Effect of MC (metronidazole: 8 mg/L, clarithromycin: 0.5 mg/L) on cell viability after co-cultured with Helicobacter pylori

Cytotoxicity for GES-1 cells exposed to *H. pylori* and MC was determined by percentage of lactate dehydrogenase (LDH) leakage and viable cell counts using trypan blue assay. The supernatant and remaining GES-1 cells were collected after incubation with *H. pylori* at MOI=100 with or without MC treatment for 24 h. LDH detection was performed according to the instruction manuals, and cell viability was investigated by counting trypan blue-negative cells. The results in Fig. S2 indicated that *H. pylori* infection reduced the cell survival rate and increased the LDH release, whereas no obvious alteration was observed after MC treatment. Moreover, cell viability and LDH release were comparable after MC treatment, indicating the non-toxic effect of MC at this dose.

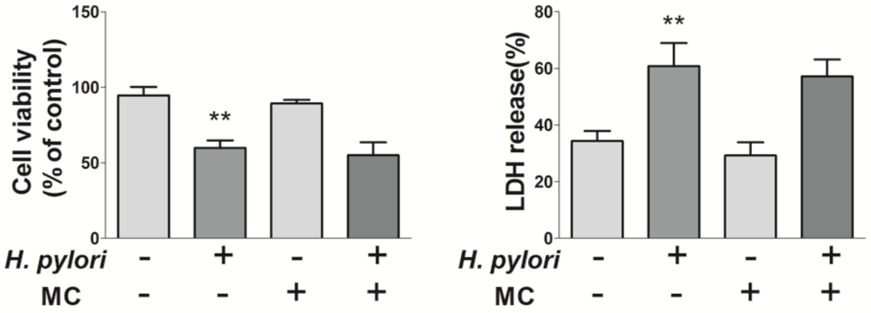

Supplementary Figure 2. Cell viability and LDH release in GES-1 cells treated with MC (n=3).
